# Service-Learning, Movies, and Infectious Diseases: Implementation of an Active Educational Program in Microbiology as a Tool for Engagement in Social Justice

**DOI:** 10.3389/fmicb.2021.589401

**Published:** 2021-06-29

**Authors:** M. Linares, N. López-Ejeda, P. Álvarez, E. Culebras, E. Díaz, M.T. García, C. Majano, M. L. Morales, A. Rodríguez-García, I. Rodríguez-Avial, C. L. Utrilla, M.V. Valenzuela, M. J. Valderrama

**Affiliations:** ^1^Department Biochemistry and Molecular Biology, Faculty of Pharmacy, Universidad Complutense de Madrid, Madrid, Spain; ^2^Department of Translational Hematology, Instituto de Investigación Hospital 12 de Octubre, Madrid, Spain; ^3^Department of Biodiversity, Ecology and Evolution, Faculty of Biology, Universidad Complutense de Madrid, Madrid, Spain; ^4^Department of Cellular Biology, Faculty of Medicine, Universidad Complutense de Madrid, Madrid, Spain; ^5^Hospital Clínico San Carlos, Madrid, Spain; ^6^Higher Technical School of Telecommunications Engineering, Universidad Politécnica de Madrid, Madrid, Spain; ^7^Department of Genetics, Physiology and Microbiology, Faculty of Biology, Universidad Complutense de Madrid, Madrid, Spain

**Keywords:** social justice, infectious diseases, microbiology, service-learning, educational methodology

## Abstract

Service-Learning is an educational methodology that allows student learning while addressing community needs. A program in microbiology and infectious diseases was implemented in Universidad Complutense de Madrid, Spain. University lecturers, clinical microbiologists, doctorate students, and undergraduates from several Bachelor Degrees and courses worked in an interdisciplinary team along with social institutions that attend disadvantaged persons. Using commercial movies that deal with infectious diseases, the students learn clinical microbiology, prepare divulgation materials, visit social centers to accompany, and help others to know about illnesses and prevention. The program was developed through two academic years and involved 58 voluntary students, 13 teachers and tutors, and 4 social entities as community partners. Postsurvey evaluation of the program revealed a highly satisfactory achievement of goals: acquiring scientific and personal competencies by university students, including critical analysis and science diffusion, solving problems or collaborative team working, and contributing, together with the tutors, to the social responsibility of the university.

## Introduction

Disadvantaged or underserved persons have the same infectious diseases as the general population, although they are disproportionately affected by higher rates of acute and chronic illnesses (Grief and Miller, 2017). Several studies in different developed countries recorded an almost 10-fold higher incidence of HIV infection, tuberculosis, and other respiratory infections, or Hepatitis B and C in homeless people when compared to general populations (Sivic et al., 2013; Tornero et al., 2016; Grief and Miller, 2017; Rudge et al., 2018; Tsai and Wilson, 2020). Other common diseases are skin infections, such as scabies, pediculosis, or tinea, and foot disorders like impetigo or cellulitis (Raoult et al., 2001; Sivic et al., 2013). A lack of hygiene, person-to-person and bedding contact, injuries, and additional problems such as drug or alcohol addiction and mental disorders are some of the reasons for this situation. Similarly, infections and sexually transmitted diseases (STD) affect other groups at risk of social exclusion, i.e., sex workers, intravenous drug addicts, immigrants, or imprisoned (Tornero et al., 2016; Grief and Miller, 2017; Wirtz et al., 2018; Franco-Paredes et al., 2020). In Spain, a study of immigrants showed that two-thirds suffered STD, including adolescents (Pérez-Morente et al., 2019), while a second one on homeless persons presenting themselves in hospitals revealed that they die 23–24 years earlier than the general population, and that infectious diseases were the second highest cause of death (Tornero et al., 2016). Additional factors that contribute to the high incidence of infectious diseases are low access to healthcare facilities and little information about proper prevention practices. There is no doubt that global interventions on specific diseases have a significant effect in reducing illness prevalence, particularly in the field of infectious diseases. However, it seems to be clear that the impact of many general approaches, without adequate local financial and human resources, may be limited (Beaman et al., 2018). Broadening the concept of health systems as defined by the World Health Organization (WHO, 2007), university members, teachers, and students together with off-campus partners could collaborate to promote heath at local level through well addressed interventions.

The methodologies used for teaching microbiology and infectious diseases in higher education have changed over the past few decades. Several initiatives that promote active student participation, rather than passive attendance at theoretical lecturers, such as clinical-case analysis, project-orientated laboratory work, external stage, or clinical practice (Desoubeaux et al., 2014; Horak et al., 2015; Merkel, 2016) have been designed with genuine student-centered learning objectives. Service-Learning (S-L) is a form of experiential education in which students engage in activities that address human and community needs together with structured opportunities intentionally designed to promote student learning (Jacoby, 1996). Learning objectives should be clearly connected to student curricula, and service activities should focus on real problems in the community. Beyond the acquisition of specific knowledge, additional benefits for students include achieving general competencies and skills that are more difficult to gain with traditional lecturer-centered methodologies, such as team working, decision-making, critical thinking, practical use of learned concepts, and even professional practice in attending actual problems of the society. Moreover, S-L constitutes an opportunity to contribute to the social function of the University, the others being education, research, and innovation (Klemenčič et al., 2020). In this sense, social responsibility can be open to general problems of society or focused on local community interests, or, moreover, specifically addressed to disadvantaged persons outside the university campus. Social justice can be defined as the objective of creating a fair and equal society in which every individual matters (Park, 2007), and some authors described that as a result of S-L experiences, students can make positive changes toward social justice and equality of opportunities (Einfield and Collins, 2008). Specifically, certain S-L projects focused on disadvantaged persons could be considered to have a social justice orientation (Lucman, 2020).

Service-Learning is more commonly used in education or social sciences, followed by healthcare areas (Stewart and Wubbena, 2014; Opazo et al., 2016; Dombrowsky et al., 2019), and less extended in basic or applied sciences (Santas, 2009; Begley, 2013). Recently, interesting S-L experiences related to microbiology courses have been described in several universities (reviewed by Webb, 2017) and many of them are based on teaching general microbiology to elementary or secondary pupils and the general population through hands-on demonstrations or small research projects (Abrahamsen, 2004; Vrentas et al., 2011; Mika et al., 2012; Webb, 2016; Valderrama et al., 2018; Groot et al., 2019).

A key step in designing S-L projects is evaluating resources, in terms of materials, experience, and time of teachers and potential students. After a pilot experience on the use of cinema in a clinical microbiology and parasitology course (García-Esteban, 2014), the Faculty of Biology, Universidad Complutense de Madrid, created a collection of more than 60 commercial films related to infectious diseases, and some lecturers have introduced film forum sessions in their classes. The utility of cinema as a teaching methodology is well known, and it has been used at different educational levels or for scientific divulgation to the general population (Darbyshire and Baker, 2012). Inspired by some experiences of Public Health S-L with a social justice orientation (Larios-Sanz et al., 2011; Behar-Horenstein et al., 2015; Sabo et al., 2015), the idea of using films for offering information on disease prevention seemed to be an appropriate framework for an S-L project. In addition to healthcare, contact and company are significant needs of disadvantaged populations that could be attended through cinema activities as well.

In this work, we describe the implementation of an S-L program, based on microbiology and infectious diseases at Universidad Complutense de Madrid, Spain, with students from Bachelor Degrees in Biology, Biochemistry, and Pharmacy. Our project, named *Movies and Infectious Diseases*, was incorporated in an initiative to promote S-L in higher education, together with the six public universities in the Madrid region and with Madrid City Council, with the objective of engaging university students with the demands of the local environment.

## Materials and Methods

A full development of an S-L project includes: (1) detecting a necessity in the community, (2) planning activities to attend to this need, (3) carrying out the activities, (4) evaluation and assessment, and (5) celebration and diffusion. Prior to designing the activities, the objectives of learning and service should be clearly defined, and notably, the learning objectives should be linked to student curricula. In addition, tutor abilities and formation, as well as university resources, need to be considered. The organization and activities of the project are described in the next sections.

### Detection of Community Needs and Targeted Populations

Increased exposure to pathogen transmission and lack of proper information about infectious diseases and prevention, as well as a need of attention and company, were detected as necessities in some groups of people in disadvantaged socioeconomic conditions and/or suffering social exclusion in Madrid, Spain, region. The targeted populations included the homeless, prisoners, drug addicts, immigrants, and teenagers from marginal areas.

### Planification of Activity

#### Potential Students and Curricular Links

The project was offered to undergraduate students of Bachelor Degrees in Biology, Biochemistry, and Pharmacy, in which they follow courses in Microbiology, Clinical Microbiology, Infectious Diseases, or Public Health. It was a voluntary activity, and the students could obtain optional academic credits.

#### Service-Learning Team and Resources

A total of 13 university members participated in the project along two editions (two academic years): four faculty lecturers, two hospital microbiologists, one postdoctoral associate researcher, three doctoral students, two young alumni, and one undergraduate. Ten social centers, coordinated by four non-governmental organizations (NGO), collaborated as community partners. Funding was obtained from the university through Innovation and Service-Learning Calls.

A collection of commercial films that include one or several infectious diseases or related subjects was available through the Universidad Complutense library. When new films were needed, bibliographic cinema resources were used (*Journal of Medicine and Movies*, Universidad de Salamanca, Spain; *NoticiaSEM*, Spanish Society for Microbiology; *Internet Movie Database*, iMDB) and films were obtained through suitable online resources (Amazon Prime, Filmin, HBO, and Netflix). A list of selected films used in the project is provided as Supplementary Material.

#### Learning and Service Objectives

Based on the principle that educational programs should be student-centered and following main S-L focuses, in the first place, learning and service objectives were designed and, in the second place, a set of activities was proposed to achieve those objectives. Table 1 summarizes the expected learning outcomes and the service aims related to the main activities of the project.

**TABLE 1:** Learning objectives for undergraduate students related to programed project activities and service objectives addressed to target population.

Learning objectives	Main activity linked	Service objectives
*Specific knowledge* – Infectious diseases and their prevention and control	– Formative sessions – Preparation of scientific information and documents	Information about infectious diseases and their prevention to disadvantaged persons
*Scientific skills* – Search for scientific information – Critical analysis – Elaboration of scientific documents – Oral expression, divulgation	– Analysis of films – Preparation of materials for the activity – Explanatory sessions at social centers – Debate within the group – Participation in conferences or divulgation events	
*General competencies* – Team work and coordination – Task responsibility, self-independent work – Adaptation to new situations	– All – Coordination of team activities and with social centers	Company and dialog
*Social and community engagement*	– Detection of community needs – Visits to social centers	

### Action

**FIGURE 1:**
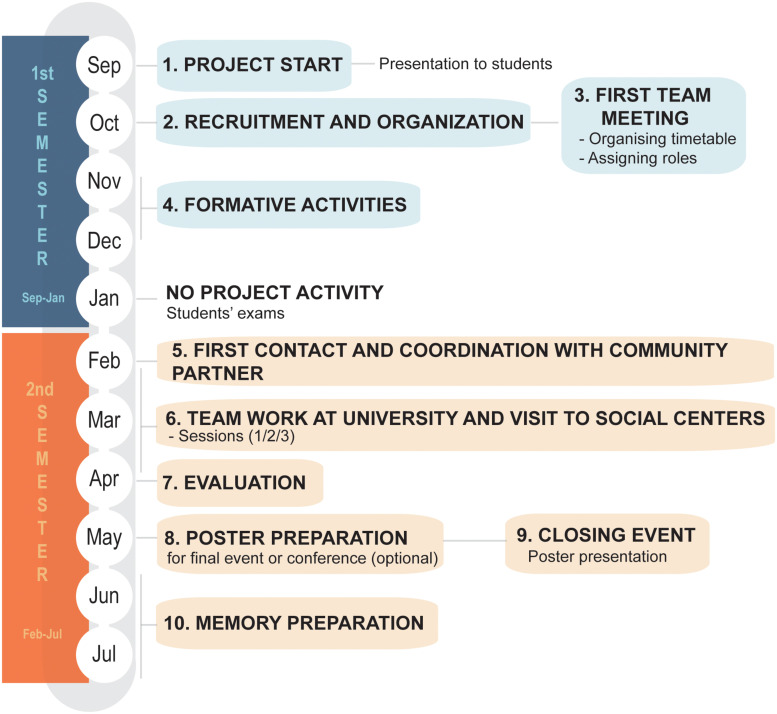
Timeline of *Movies and Infectious Diseases* Serving-Learning (S-L) project at Universidad Complutense de Madrid, Spain, along a single academic year.

The activities along an academic year are presented in Figure 1, and the description in the text will follow the numbers used in the diagram.

#### Presentation, Student Recruitment, and Group Organization

The project was introduced to the undergraduates by lecturers during the first weeks of the academic course. Volunteers were organized into groups of four to five members, supervised by two tutors, and a social center was assigned to each group. The teams had a start-up meeting for self-organizing and assigning roles. Although the members participated in all the stages, one person was responsible for streamlining each activity: (a) team coordination and communication with tutors and community partner, (b) preparation of scientific and divulgation materials, (c) film selection and analysis, and (d) surveys undertaking and celebrations. Tutors and undergraduates worked at the same level with a non-hierarchical, horizontal, coordination.

#### Formative Sessions

The students received formative talks, organized in a relaxed and participative format, about the main subjects of the project: (1) infectious diseases, with a clinical microbiology analyst, (2) community needs and social exclusion, conducted by a NGO coordinator, and (3) cinema and medicine, accompanied by a filmmaker.

#### Teamwork and Visits to Community Partner Centers

Each team visited their assigned social center on three occasions and worked at the university to prepare the sessions. At all times, the university students were accompanied by the academic tutors and by the coordinators of the centers, following the recommended access procedures. Previously, the coordinator student and tutors had an initial visit to know about the characteristics of the center and persons attended.

In session 1, the students conducted a dialog with the group in order to detect their interests and needs. They suggested diseases, posed, or answered questions, and finally selected one infectious illness among the different suggestions. When back in the university, the students searched for films related to the selected disease, focusing on the adequacy for the social center. Aspects such as duration, drama or action, or how the illness was treated in the film were discussed in order to select the most suitable one. Next, they critically analyzed the film, investigated the infectious disease, summarized the results in a common template, and prepared divulgation materials. A question–answer session was organized for which the students prepared putative questions that could be asked by participating persons and practised answering these using clear and correct, non-technical, scientific language.

In session 2, the film was projected in the social center, with popcorn and refreshments. Afterward, a relaxed and participative dialog was opened with the aim of resolving doubts and reinforcing key messages about infectious illnesses and prevention.

During session 3, the last one in the social centers, a voluntary survey was presented to participants, during a small celebration party, where the colloquium about infectious illnesses would continue.

### Evaluation and Assessment

Assessment of the project and activities was performed using post-service surveys administered anonymously, with a Likert response 1–5 scale (1, very low/strongly disagree; 5, very high/strongly agree). All the university students and tutors completed a set of 60 questions, prepared with an online Google Forms tool, that were organized into six sections: (1) scientific competences and learning, (2) competence and skill acquisition, (3) service and social consciousness, (4) S-L methodology, (5) reasons for participating, and (6) activities and general satisfaction. For the social centers, a brief survey was completed on a voluntary basis by persons who attended and coordinators, with questions about the activity, film projection, or explanations of the students. All the surveys included blank spaces for free redaction to collect qualitative impressions, where “the best” or “the worst” of the project could be pointed out. At the end of the project, those students who wanted to obtain optional academic credits elaborated a three-page reflection paper.

Statistical analysis of the results was performed using the GraphPad Prism Software (version 7). The non-parametric Mann–Whitney or Kruskal–Wallis tests were used for comparisons between two or more groups. For multiple comparisons between groups, Dunn’s test was performed. For this, *p* values < 0.05 were considered statistically significant.

### Celebration and Diffusion

A closing event was organized at the university with all the students, tutors, and coordinators of the community partner centers where the groups of students explained their work using a poster presentation. Additionally, supervised by their tutors, some students presented their S-L experience to scientific or divulgation conferences.

## Results

### Participants and Academic Activities of the S-L Project

A total of 58 voluntary students participated in the project along two academic years: Biology, 4th year = 16; Biochemistry, 3rd year = 28; Biomedical Engineering, 2nd year = 1; Pharmacy 2nd year = 11; and Pharmacy, 5th year = 2. They were organized in multidisciplinary teams of different courses and years, which prompted coordination and collaborative learning.

In accordance with the characteristics and interests of the group of persons at the social centers, one or more infectious illnesses, or related aspects, as well as a suitable film were selected by students (Table 2).

**TABLE 2:** Selected films about infectious diseases used in the Service-Learning (S-L) project.

Collective	Subject*	Selected film
Homeless, prisoners	STDs, AIDS	*Boys on the side*, 1995 *Dallas Buyers Club*, 2013 *La vida alegre*, 1987 *Philadelphia*, 1993 *Yesterday*, 2004
Homeless	Vaccines	*I am legend*, 2007
Teenagers, immigrants	Pandemic	*Contagion*, 2011 *World War Z*, 2013
Homeless	VBD	*Der Medicus*, 2013

*STDs, sexually transmitted diseases; AIDS, acquired immune deficiency syndrome; VBD, vector-borne diseases.

The results of the analysis of the films were presented in two types of files: (A) *Scientific card*, which included 1. Name of the disease, 2. Causal agent/s, 3. Epidemiology (transmission, incidence, prevalence), 4. Symptomatology, 5. Diagnosis, 6. Treatment, 7. Prevention, including vaccination; and (B) *Cinematographic card*, with all the sequences related with the illness (minutes and seconds recorded) grouped in the same sections as the scientific document and including an analysis of scientific errors, when applicable. These detailed documents were used by the students as supporting material for the colloquium that was undertaken after the film projection in the social centers. Additionally, the students prepared a range of divulgation materials, such as brochures, posters, photographs or pictures, sanitary resources, and short videos, as well as entertaining games, such as Trivial or Bingo games, to accompany the discussion sessions.

### Assessment of Utility of the S-L Strategy on Students and Community

**FIGURE 2:**
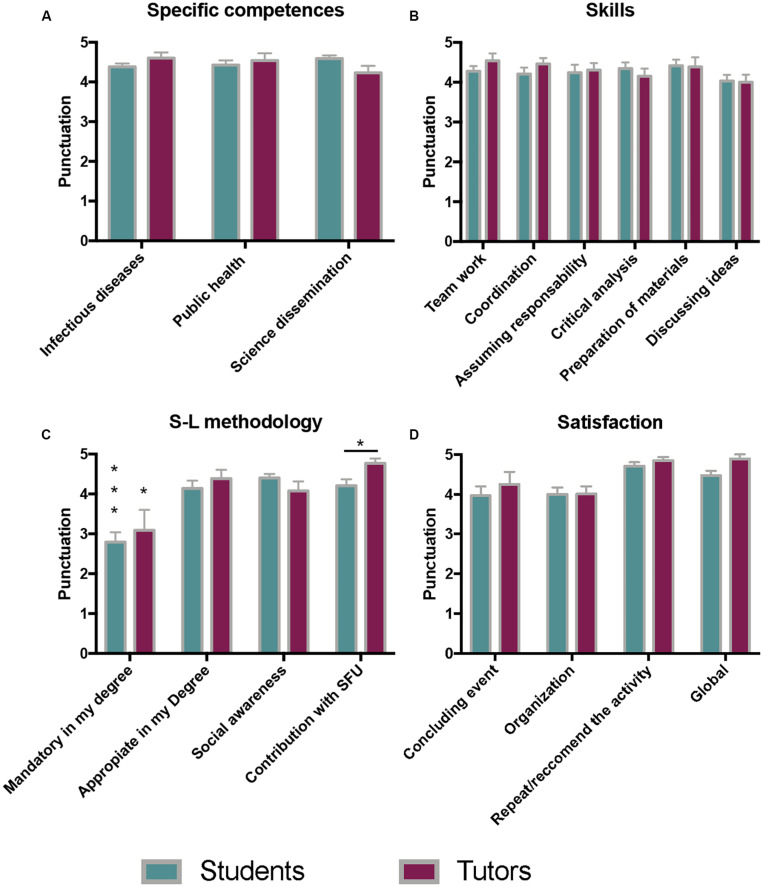
Selected survey results from undergraduate students (*n* = 29) and tutors (*n* = 13) involved in *Movies and Infectious Diseases* S-L project at Universidad Complutense de Madrid, Spain, after 2 years of experience. **(A)** Scientific competencies and learning. **(B)** General skills acquired during the activities. **(C)** Adequacy and utility of the S-L methodology, **(D)** Satisfaction with the experience and activities. All data are expressed as mean and standard deviation on a 1–5 scale (1, very low/strongly disagree; 5, very high/strongly agree). Significant differences between the marked groups and the remaining groups are highlighted with asterisks (*p < 0.05; ***p < 0.0005).

**FIGURE 3:**
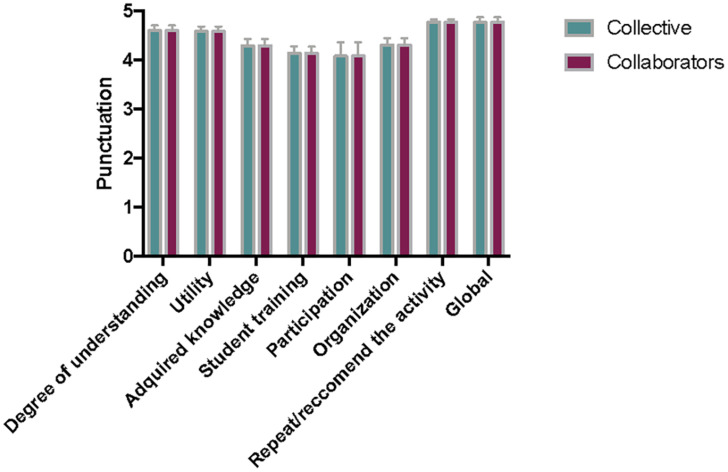
Selected survey results from community partners (collective, *n* = 54; collaborators, *n* = 7) involved in *Movies and Infectious diseases* S-L project at Universidad Complutense de Madrid, Spain. All data are expressed as mean and standard deviation on a 1–5 scale (1, very low/strongly disagree; 5, very high/strongly agree).

Significant results from the post-experience surveys are presented in Figure 2 for students (*n* = 29) and tutors (*n* = 13). The data of the two editions of the project are included and analysed together. Figure 3 shows the results for community partners (persons attended, *n* = 54; social centers coordinators, *n* = 7) and correspond only to the first edition, as the visits to the social centers were not completed in the second edition of the project (2020) due to restrictions on movement and meetings arising from the coronavirus (COVID-19) pandemic. Mean values out of 5 and standard deviations were calculated from the 1–5 options of the scale.

#### Active Learning

According to the results of students and tutors’ surveys, the benefits of the S-L experience were centered around acquiring or reinforcing specific knowledge about infectious diseases (4.4/5) and competences in public health (4.4/5) or science dissemination (4.6/5) (Figure 2A). In addition, improvement in personnel and scientific skills was also recognized (Figure 2B), i.e., teamwork and coordination (4.3 and 4.2 over 5, respectively), discussion of ideas and critical analysis (4.2/5), and preparation of divulgation materials (4.4/5). The evaluation of the same aspects made by the tutors rendered similar results, with no significant differences encountered (Figures 2A,B). Individual investigation about pathogens and diseases, film analysis and group discussions, and answering questions or explaining microbiology to non-specialized people were highlighted by students in the spaces for free writing of the surveys, as the best activities for learning. Interaction with team partners, including tutors and colleagues, was also considered as adequate for learning and preparation for future professional work (mean value 4,3/5, data not shown in Figure 2). Most of the participants thought that, although sometimes it was difficult to coordinate different timetables and personal schedules, the heterogeneity of team members was enriching as they could learn from each other (written opinions from surveys).

#### Serving Disadvantaged Persons

Evaluation of service satisfaction was achieved through a survey of community partners. The results (Figure 3) showed high scores for the utility of S-L activities for understanding and acquiring knowledge about infections and how to prevent them (mean of three items for attended persons and coordinators, 4.5/5), thanks to the students’ preparation and adequate answers to the questions asked by the collectives (collective 4.9/5, coordinators, 4.7/5). In the written opinions, the disadvantaged persons appreciated most profoundly the company, respect, attention, and equal treatment of the students and tutors. The data presented in Figure 3 correspond only to the first edition, as the visits to the social centers were not completed in the second edition of the project in 2020, as previously mentioned. Nevertheless, the coordinators of NGOs, who participated in the online closing event where the students presented their work (9, Figure 1), highlighted the usefulness of the activity and the materials prepared by the students for divulgation at their centers (data not shown).

#### Service-Learning as an Efficient Educational Methodology at the University

The motivations of the university students for choosing the *Movies and Infectious Diseases* S-L project were (in order of punctuation) social involvement (4.6/5), the possibility to acquire general competences and skills (4.5/5), curiosity (4.2/5), and strengthening of academic knowledge of clinical microbiology (4.1/5). In comparison, earning elective credits and occupying their time were not important motivations (2.7 and 2.2, respectively) with significant differences (*p* < 0.05 and *p* < 0.0005, respectively).

Undergraduates and tutors thought that S-L methodology was adequate for their degrees, increased their social awareness, and contributed to the social responsibility of the University (4.0–4.8). Nevertheless, although many of them described the activity as enriching and directly applicable to their future work in clinical microbiology, public health, or related areas (written opinions in surveys), most of them considered that S-L should not be mandatory within microbiology courses (mean value of teachers and students, 2.8/5 and *p* < 0.05). The data of S-L methodology are shown in Figure 2C.

Finally, global satisfaction of the participants was used to measure the success of the project and, notably, high scores were obtained (4.5/5 students and 4.6/5 tutors, in Figure 2D; 4.8 collective and 4.6 coordinators in Figure 3). Most of the participants would join again and recommend the S-L project to companions (4.8 and 4.7 out of 5 for students and teachers, respectively, Figure 2D; 4.8 for persons and coordinators in social centers, Figure 3).

## Discussion

Service-Learning initiatives specifically focused on clinical microbiology are usually included as a part of Public Health actions for Nursing or Medicine courses (Larios-Sanz et al., 2011; Abu-Shakra, 2012; Cain, 2013; Stewart and Wubbena, 2014), but they are less used in Bachelor Degrees in Sciences. The S-L program described in this work was developed with students and university tutors of Biology, Biochemistry, and Pharmacy, as these degrees include courses on Clinical Microbiology, and this would indicate the usefulness of this type of S-L programs in different university careers. Similarly, another S-L experience was carried out in Universidad Complutense de Madrid, based on antibiotic resistance, in which the Faculties of Biology, Pharmacy and Veterinary were involved (Valderrama et al., 2018). To our knowledge, cinema had not been applied in S-L experiences in clinical microbiology (Webb, 2017), although it is frequently used as a tool for teaching infectious diseases (García-Sánchez et al., 2002). The results of our project clearly showed the utility of commercial films in the student learning process at the university level and the importance of attending to community needs.

Concerning the learning objectives, specific knowledge about infectious diseases and public health were significant achievements according to the results of the surveys for students and tutors. As only post-experience surveys were carried out on the students, no data were available to compare scientific knowledge before and after the activity. However, in some studies, it was found that final grades of students who participated in an S-L project and those who followed traditional lecturers in clinical microbiology were similar (Cain, 2013). Most frequently, specific learning outcomes through S-L are not quantified in terms of global academic marks but the progress is appreciated by the students themselves and their teachers (Abu-Shakra, 2012; Valderrama et al., 2018). The data from the student surveys did not show a significant difference between acquiring new concepts and reinforcing previous ones, probably because most of the participants had studied a clinical microbiology course before joining the project and they had already acquired some background knowledge. Rather than being a pitfall, it is considered that strengthening of learned concepts by applying them to real situations is one the goals of S-L (Cain, 2013). Critical analysis of the films was highlighted in the surveys as a very adequate method for learning, as has been previously described when using cinema to other health areas (Weerts, 2005). Nevertheless, a literature review of the use of commercial films as a teaching resource for health sciences students concluded that there are few studies that quantify the increase in learning to clearly demonstrate the usefulness of the methodology, beyond the subjective perception of students and lecturers (Díaz et al., 2015).

In our project, students from different degrees and academic levels worked in mixed groups that included juniors (2nd year) and some seniors (4th or 5th year) and the heterogeneity was pointed out in the surveys as an ideal form of learning from teammates. This benefit was previously observed in several collaborative learning courses in microbiology, where junior students were helped by more experienced ones (Rutherford, 2015; Hou et al., 2018). Moreover, it has been described that the undergraduates do not need to have a high level of specific knowledge to participate, although they will require more supervision by their tutors (Dombrowsky et al., 2019).

Salient benefits of S-L on students’ academic progress are related to skills acquisition, as can be concluded from our results. Competence on explaining microbiology to non-experienced people, answering questions, and clarifying doubts about infections, transmission of pathogens, or prevention procedures can be highlighted. In the same sense, learning by teaching others has been described as an efficient methodology in microbiology and other disciplines (Rutherford, 2015; Webb, 2016, 2017). Additionally, team-working ability, coordination, assuming responsibilities or leadership, and problem solving and adaptation were freely expressed by the students in their surveys as significant goals, and similar results have been described for other S-L programs (Opazo et al., 2016; Valderrama et al., 2018).

Assessing Service-Learning projects should not only focus on student learning, and the community service must be also evaluated. The high level of satisfaction of social partners confirmed the usefulness of the *Movies and Infectious Diseases* program for addressing real needs of outside-campus communities. Although it might be difficult to assess service outcomes through quantitative surveys, it is recognized that the results obtained from post-experience reports, oral opinions, and group discussions are also valuable (Holland, 2001). In our S-L experience, social awareness and engagement were the main motivations and satisfaction elements for students and tutors, as is characteristically described for this kind of projects (Bringle and Clayton, 2012). As for the students, the most repeated positive impressions (marked as “the best” in the surveys) were “*discovering social situations unknown until then*,” “*meeting disadvantaged persons*,” and “*sharing their scientific knowledge with others in need*.”

In summary, the results of the program “*Movies and Infectious Diseases*” illustrate that S-L fulfils the objective of active learning of university students on clinical microbiology courses while attending community needs, i.e., helping to prevent infections to disadvantaged persons. We understand that the S-L program implemented is an excellent strategy from an academic point of view, which promotes not only scientific knowledge but, almost most importantly, critical thinking, teamwork, and responsibility. Moreover, the connection with the real needs of the community prompted personal and group engagement in social justice in both university students and tutors.

## Data Availability Statement

The original contributions presented in the study are included in the article/Supplementary Material, further inquiries can be directed to the corresponding author.

## Ethics Statement

The studies involving human participants were reviewed and approved by Ethics and Deontology Committee. University Complutense of Madrid (signed by F. J. M. Fernández Vallina, President of the Committee). Written informed consent to participate in this study was provided by the participants’ legal guardian/next of kin. Written informed consent was obtained from the individual(s) for the publication of any potentially identifiable images or data included in this article.

## Author Contributions

MJV: conceptualization. PÁ, EC, ED, MTG, NL-E, ML, CM, MLM, AR-G, IR-A, CLU, MJV, and MVV: experimental procedures. ML, NL-E, and MJV: data analysis and interpretation. MJV: the manuscript preparation. All authors have read an agreed this version of the manuscript.

## Conflict of Interest

The authors declare that the research was conducted in the absence of any commercial or financial relationships that could be construed as a potential conflict of interest.
