# Supplementary material for: Service-Learning, Movies, and Infectious Diseases: Implementation of an Active Educational Program in Microbiology as a Tool for Engagement in Social Justice
Source: Front Microbiol. 2021 Jun 29;12:589401. doi: 10.3389/fmicb.2021.589401 (PMC8276174; doi:10.3389/fmicb.2021.589401)
Supplement: Supplementary file 1 [file Data_Sheet_1.pdf]

Supplementary material

Selected films about infectious diseases used in the S-L project at Universidad Complutense de Madrid, Spain

Title	Director	Year	Topic
12 monkeys	Terry Gilliam	1995	vaccines, pandemic
Albert Nobbs	Rodrigo García	2011	typhoid fever
All about my mother	Pedro Almodóvar	1999	STIs
And the band played on	Roger Spottiswoode	1993	AIDS
Arachnophobia	Frank Marshall	1990	arthropods
Awakenings	Penny Marshall	1990	encephalitis
Barefoot in the park	Neil Simon	1967	flu
Bohemian Rapsody	Bryan Singer	2018	AIDS
Boys on the side	Herbert Ross	1995	AIDS
City of Joy	Roland Joffé	1992	leprosy
Contagion	Steven Soderbergh	2011	pandemic
Dallas Buyers Club	Jean-Marc Vallée	2013	AIDS
Home alone 3	Raja Gosnell	1997	varicella
I am legend	Francis Lawrence	2009	vaccines
In enemy hands (U-Boat)	Tony Giglio	2004	meningitis
In love and war	Richard Attemborough	1997	infection (skin)
Little women	Gillian Armstrong	1994	scarlet fever
Love in the time of cholera	Mike Newell	2007	cholera
Mission: Impossible 2	John Woo	2000	pandemic
Moulin Rouge	Baz Luhrmann	2001	tuberculosis
Nowhere in Africa	Caroline Link	2001	malaria
Out of Africa	Sydney Pollack	1985	syphilis, malaria
Outbreak	Wolfgang Petersen	1995	pandemic
Pandemic: how to prevent an outbreak	Doug Shultz	2020	pandemic
Pasteur y Koch: a duel of giants in the war of microbes	Matthiew Schwarz	2018	vaccines
Philadelphia	Jonathan Demme	1993	AIDS
The Andromeda Strain	Robert Wise	1971	pandemic
The children of Huang Shi	Roger Spottiswoode	2008	tetanus, lice, malaria
The Constant Gardener	Fernando Meirelles	2005	tuberculosis
The green mile	Frank Darabont	1999	infection (urine)
The happy life	Fernando Colomo	1987	scarlet fever

The horse soldiers	John Ford	1959	mumps
The horseman on the roof	Jean-Paul Rappeneau	1995	cholera
The motorcycle diaries	Walter Salles	2004	leprosy
The painted veil	John Curran	2006	cholera
The Physician	Philipp Stölzl	2013	pestilence
The reader	Stephen Daldry	2008	scarlet fever
The story of us	Rob Reiner	1999	varicella
The witches of Eastwick	George Miller	1987	HSV
Tombstone	George Cosmatos	1993	tuberculosis
Wolf	Mike Nichols	1994	rabies
World War Z	Marc Foster	2013	vaccines, pandemic
Yesterday	Danny Boyle	2019	AIDS

STI: sexually transmitted infections; AIDS: Acquired immunodeficiency syndrome; HSV: Herpes simplex virus.
