# Supplementary material for: Service-Learning, Movies, and Infectious Diseases: Implementation of an Active Educational Program in Microbiology as a Tool for Engagement in Social Justice
Source: Front Microbiol. 2021 Jun 29;12:589401. doi: 10.3389/fmicb.2021.589401 (PMC8276174; doi:10.3389/fmicb.2021.589401)
Supplement: Supplementary file 2 [file Data_Sheet_2.pdf]

Movies and Infectious Diseases S-L project
Post-survey for students of two editions. Questions and results

0. Information on the survey and agreement on confidentiality terms

Values: 1-5: 1, very low/strongly disagree - 5, very high/strongly agree	**Mean values**
**1. Contribution to learning and aquisition of specific competencies**	
The project is related to my studies	4,59
I have learned new concepts about infectious diseases	3,93
I have reinforced previous concepts I had studied during my bachelor degree	4,61
I have learned about Public Health as a profesional activity	4,14
I understood the relevance of Public Health	4,71
The project increased my perception about the importance of scientific divulgation	4,71
I practised correct scientific expression	4,46
The experience has contributed to orientate my future career	3,57
**2. Contribution to develop general competencies and skills**	
Teamwork	4,28
Coordination	4,21
Selforganization and managing my time	4,28
Assuming responsability	4,24
Critical analysis	4,34
Preparation of scientific and divultation materials	4,41
Oral expression	4,00
Solving doubts	4,11
Expressing my own ideas	4,00
**3. Contribution to community service and to my social conciousness**	
I did a service to community	4,55
I think I did normal volunteering*	3,60
My social conciousness has increased	4,66
I discovered social disadvantaged situations, previusly unknown for me	4,03

I kew the Social Function of the University (SFU)	2,31
I have contributed with the SFU	4,31

4. S-L Methdology

I knew S-L	1,86
I had participated in S-L projects before	1,32
I have participated in other projects of Innovative Learning metodologies	1,71
S-L is an adequate methdology ofr learning specific contents related to my bachelor degree	4,41
S-L is adequate for developong general competencies that are not aquired with normal lectures	4,37
The activity could be inlcuded as mandatory in one of the subjects I am studying	2,79
S-L shwould be mandatory at least once in all the university studies	4,28
I get information about S-L projects from my faculty or from the university	2,52

5. Reassons to particiate in the project

Occupying my time	2,10
Obtain extra credits	2,69
Improve muy curriculum	4,07
Learn scientific contents	4,21
Improve my general competencies	4,45
Confront a personal or social challenge	4,55
Collaborate to solve problems	4,69
Help others	4,72
Curiosity	4,24

6. Development of the project and activities and Global satisfaction

Formative session: Sexually Transmitted Diseases	4,56
Formative session: Cinema	4,28
Formative session: Social needs	4,38
Selection of infectious illness and films	4,26
Analysis of films	4,14
Preparation of cientific and cinematografic cards	4,37
Preparation of divulgation materials	3,86
Question-answer session	3,90

Number of sessions at social centres*	2,50
Film projection at social centre*	3,44
Colloquium at social centre*	3,78
Poster elaboration	4,42
Final session	4,04
General organization	4,03
Time requiered	3,90
Number of formative sessions	4,32
Number of team meetings	4,14
Face-to-face meetings	4,37
Visits to social centres*	3,40
Virtual meetings	4,42
Interactions with team mates	4,34
Interaction with tutors	4,10
Interaction with collective at social centre*	4,60
Interaction with coordinators at asocial centre*	4,10
Would you participate again?	4,59
Woiuld you recommend the activity?	4,69
Golbal satisfaction with the activity	4,55

* Results correspond only to one edition of the project

7. Questions for free writting (written opinions not included due to their extension)

Which part of the project was more difficult?
Which part of the project was more useful for your learning?
The best of the project
The worst of the project
Suggestions for improving
Free coment on what the project has brought you personally

Movies and Infectious Diseases S-L project
Post-survey for tutors of two editions of the project. Questions and results

Information on the survey and agreement on confidentiality terms

Values: 1-5: 1, very low/strongly disagree - 5, very high/strongly agree	**Mean values**
**1. Contribution to learning and aquisition of specific competencies by the students**	
The project is related with the students' curricula	5,00
The students have aquired new concepts about infectious diseases	4,38
The students have reinforced previous concepts	4,62
The students understood the relevance of Public Health	4,54
The students apreciated the importance of scientific divulgation	4,38
The students improved their scientific expression	4,00
**2. Contribution to develop general competencies and skills by the students**	
Teamwork	4,62
Coordination	4,38
Assuming responsability	4,38
Preparation of scientific and divulgation materials	4,46
Critical analysis	4,15
Oral expression	3,92
Solvimg doubts	4,00
Explaining science to non-specialised public	4,33
**3. Contribution to community service and to my social conciousness**	
My social conciousness has increased	4,46
I discovered social disadvantaged situations, previusly unknown for me	4,08
I did a service to community	4,46
I think I did normal volunteering	1,77
**4. S-L Methdology**	
I knew S-L	3,69

I had participated in S-L projects before	3,00
I have participated in other projects of Innovative Learning metodologies	3,23
S-L is an adequate methdology for students	4,69
S-L shwould be mandatory at least once in all the university studies	4,46
The activity could be inlcuded as mandatory in one of the subjects I teach	2,42
S-L contributes to Social Function of the University	4,85

6. Development of the project and activities and Global satisfaction

Organisation	4,23
Time requierd	4,08
Face-to-face meetings with the students	3,54
Virtual meetings	4,25
Film projection at social centre*	4,40
Colloquium at social centre*	4,20
Formative session: Sexually Transmitted Diseases	4,88
Formative session: Cinema	4,75
Formative session: social needs	4,50
Final session	4,75
Interaction with students	4,31
Interaction with tutors	4,08
Intereation with collective at social centre*	4,00
Interaction with the coordinators at social centre*	4,20
Would you repeat the activity?	4,85
Would you reccommend the activity?	4,92
Global satisfaction	4,77

* Results correspond only to one edition of the project

7. Questions for free writting (written opinions not included due to their extension)

Which part of the project was more useful for students' learning?
The best of the project
The worst of the project
Suggestions for improving
